# Overweight and/or obesity and its determinants among under-five children in East African countries: A multilevel analysis using Bayesian approach

**DOI:** 10.1016/j.heliyon.2021.e08643

**Published:** 2021-12-20

**Authors:** Sofonyas Abebaw Tiruneh, Alemayehu Digssie Gebremariam, Melaku Tadege Engidaw, Desalegn Tesfa, Fentaw Teshome Dagnaw, Edgeit Abebe Zewde, Melkalem Mamuye Azanaw

**Affiliations:** a Department of Public Health (Epidemiology), College of Health Sciences, Debre Tabor University, Debre Tabor, Ethiopia; b Department of Public Health (Human Nutrition), College of Health Sciences, Debre Tabor University, Debre Tabor, Ethiopia; c Department of Public Health (Reproductive Health), College of Health Sciences, Debre Tabor University, Debre Tabor, Ethiopia; d Department of Public Health (Health Service Management), College of Health Sciences, Debre Tabor University, Debre Tabor, Ethiopia; e Department of Biomedical Science (Medical Physiology), College of Health Sciences, Debre Tabor University, Debre Tabor, Ethiopia

**Keywords:** Overweight, Obesity, Under-five children, East Africa

## Abstract

**Introduction:**

Childhood overweight and/or obesity become a significant public health problem in the 21^st^ century. It is a double burden next to undernutrition and has a dramatic rise in low- and middle-income countries. This study aimed to determine the prevalence of overweight and/or obesity and its determinants among under-five children in East African Countries.

**Methods:**

Data were retrieved from the recent nationally representative demographic and health survey datasets from eleven East African Countries. A total of 89,091 weighted numbers of under-five children participated. Statistical analysis was performed using the R (Brms R-package) software. Multivariable mixed-effects logistic regression analysis using the Bayesian approach was employed to identify the factors affecting overweight and/or obesity among under-five children.

**Results:**

Overall, 4.59% (95% CI, 4.45–4.73) of under-five children in East African Countries were overweight and/or obese. Under-five children overweight and/or obesity was highest in Comoros and lowest in Burundi. Under-five children aged older than two years (Adjusted odds ratio (AOR) = 0.65, 95% credible interval (CrI), 0.57–0.73), females (AOR = 0.84, 95% CrI:, 0.75–0.94), under-five children live from rich household wealth status (AOR = 1.25, 95% CrI, 1.06–1.49), under-five children living in Malawi (AOR = 2.60, 95% CrI, 1.49–4.51), Mozambique (AOR = 5.26, 95% CrI, 3.52–7.79), Rwanda (AOR = 5.63, 95% CrI, 3.46–9.08), Tanzania (AOR = 2.15, 95% CrI, 1.47–3.12), and Uganda (AOR = 2.62, 95% CrI, 1.71–3.99) were a significant determinant for under-five overweight and/or obesity.

**Conclusion:**

Overweight and/or obesity among under-five children become a problem in low and middle-income countries. Older under-five children, male sex, children who live in rich household wealth, and children who live in a country in Malawi, Mozambique, Ruanda, Tanzania, and Uganda were significantly affected by overweight and/or obesity. Therefore, in these countries, responsible stakeholders shall give primary attention to curve the alarming increase in overweight and/or obesity among under-five children.

## 1 Introduction

Childhood overweight and/or obesity has become an epidemic not only for developed nations but also for developing countries [1]. Childhood overweight and/or obesity is a double burden with undernutrition; nowadays overweight and/or obesity dramatically rise in low and middle-income countries (LMICs), particularly in urban settings [2]. Globally, 38 million under-five children were overweight and/or obese in 2019 [3]. In the African region since 2000, the burden of overweight and/or obesity among under-five children has increased by nearly 24% [4]. Overweight and/or obesity during the childhood period affect physical and psychological health with a chance to stay obese during the adulthood period. Obesity during this period might be prone to develop non-communicable diseases (NCDs) like diabetes and cardiovascular diseases [5]. Obesity also permanently increases oxidative stress. Over-expression of oxidative stress damages cellular structures and the under-production of anti-oxidant mechanisms, leading to the development of obesity-related complications [6]. Therefore, the prevention of overweight and/or obesity in the childhood period is an effective strategy to prevent chronic diseases in adults.

Non-communicable diseases have become a major problem related to overnutrition due to the rapid nutritional and epidemiological transition [7, 8]. The global prevalence of under-five obesity was 7% in 2012 and was predicted to be <11% in 2025 [9]. Even if the mechanism of obesity is not known, environmental factors, lifestyle preferences, and cultural environment play vital roles in the rising of obesity [5].

Evidence indicates that the importance of early life overweight and/or obesity management used for mitigating the risk of obesity later in life [10]. Globalization and nutrition transition improved the living standards of individuals. However, they had some negative consequences that directly or indirectly led to poor dietary consumption and physical activity patterns. Because of this, the occurrence of overweight and/or obesity among under-five children, besides diet-related chronic non-communicable diseases later in life increased persistently.

As far as we are concerned, there is no evidence regarding under-five overweight and/or obesity using the recent nationally representative demographic and health survey using a large dataset in the region. Besides, previous studies in the developing world primarily focused on under-five undernutrition. However, currently in developing countries, under-five obesity is a double burden with undernutrition and becomes a very rampant problem. Thus, documenting this evidence will help policymakers, health planners, and the community itself in the region. Besides, this study accounts for some flat prior information to identify the factors affecting overweight and/or obesity among under-five children which may help as a baseline comparison with the frequentist approach. Therefore, this study aimed to determine the pooled prevalence of overweight and/or obesity and its associated factors among under-five children in East African Countries using the recent demographic and health survey datasets.

## 2 Methods and materials

### 2.1 Data sources

In this study, eleven East African countries (namely Burundi, Ethiopia, Kenya, Comoros, Malawi, Mozambique, Rwanda, Tanzania, Uganda, Zambia, and Zimbabwe) the recent nationally representative demography and health survey datasets were used. Demographic health survey (DHS) data were collected nationally using a multistage stratified cluster sampling technique. The details of the recorded data were accessed at https://dhsprogram.com/.

### 2.2 Populations and samples

The source populations were the number of de facto living children between aged 0 and 59 months before preceding five years the survey among eleven East African Countries whereas the study populations were the number of de facto living children between aged 0 and 59 months preceding five years the survey period in the selected Enumeration Areas (EAs) in each country. For this study, the data were extracted from the personal record (PR) file from the standard DHS dataset with at least one recent survey between 2010 and 2019. A total of 89,935 under-five children were included from eleven East African Countries. The details about samples included for each country is available in table one (Table 1).

**Table 1:** Sample size for under-five mortality in east African countries.

East Africa countries	DHS year	Sample size	
		Unweighted	Weighted
Kenya	2014	20,757	19,249
Burundi	2016/17	9,697	10,513
Ethiopia	2016	6,410	6,493
Comoros	2012	2,378	2,445
Malawi	2015/16	5,790	5.814
Mozambique	2011	9,296	9,423
Rwanda	2014/15	3,825	3,858
Tanzania	2015/16	10,301	9,942
Uganda	2016	5,286	5,243
Zambia	2018/19	9,694	9,705
Zimbabwe	2015	6,164	6,406
**Total sample size**		**89,598**	**89,091**

### 2.3 Study variables

The outcome variable of this study was overweight and/or obesity among under-five children. According to the new World Health Organization (WHO) child growth monitoring charts, overweight and/or obesity is declared if the child's weight-for-height z-score is above plus 2 (+2.0) standards deviations (SD) above the mean [11]. The independent variables were individual-level variables (maternal education status, child age, and sex, preceding birth interval, birth order, child anemia status, household wealth) and region or province-level factors (residence, altitude, country, and country income per capita). Household wealth status was computed using principal component analysis by the demographic and health survey program [12]. Household wealth status was categorized in five quintiles as poorest, poor, middle, rich, and richest. Further household wealth was classified as in third quintiles as poor, middle, and rich. The demographic and health survey-7 (DHS-7) guideline classified under-five anemia as follows: No anemia if the child has hemoglobin count greater than 11 g per deciliter, any anemia: number of children whose hemoglobin count is less than 11 g/dl, mild anemia: number of under-five children whose hemoglobin count is between 10.0 and 10.9 g/dl, moderate anemia: number of children whose hemoglobin count is between 7.0 and 9.9 g/dl, and severe anemia: number of children whose hemoglobin count is less than 7.0 g/dl.

### 2.4 Data management and analysis

The data were cleaned, coded, and extracted using STATA version 16/MP software. Sample weighting was done for each country before further analysis.

### 2.5 Multilevel analysis using Bayesian approach

To identify the factors affecting under-five overweight and/or obesity, we employed multilevel mixed-effects logistic regression analysis using the Bayesian approach. The Bayesian approach credible intervals have a similar role to classical confidence intervals, but the philosophy of their composition and interpretation are quite different [13]. The Bayesian statistical approach gives the probability of incorporating additional prior information external to the data by prior distributions. This additional prior information may improve the accuracy and credibility of effect size estimations. Thus, the Bayesian statistical approach that gives a reasonable interpretation and classical confidence interval should not be applied. Therefore, the Bayesian credible interval interpretation seems intuitive and frequentist confidence intervals are misinterpreted as Bayesian credible intervals.

Region or province was the random variable. Children in the household nested within units the next higher level in the region or province of the country. We used the intraclass correlation (ICC) value > 10% to consider the variation of the child to be overweight and/or obese across regions or provinces of the country. Therefore, modeling flat models would affect the true effect size. The nutritional status of the child represented by

$$Y_{ij}=\left\{ \begin{array}{l} \text{Overweight}\;\text{and/or}\;\text{obese}\\ \text{Normal}\;\text{or}\;\text{underweight} \end{array}\right.$$

Since it is a binary data type, multilevel mixed-effects logistic regression analysis is conducted using the Bayesian approach.

The effect size of each parameter is estimated from the posterior distribution which is the distribution of both the prior information and the likelihood of the data [14]. We used improper/vague flat prior with normal distribution to estimate regression coefficients for each parameter and Cauchy distribution to estimate the variance. The *Brms* R-language uses the Hamiltonian Monte-Carlo (HMC) and its extensions No-U-Turn Sampler (NUTS) technique in Stan on the back-end. To estimate the posterior distribution, we use iteration = 2000, warmup = 1000 (number of iterations that was discarded), chains = 2, initials (the starting values of the iterations) = 0, cores (specifies the number of cores used for the algorithm) = 2 and adapt delta (controls divergent transition) = 0.95. From the four models (null model, individual variable model, region or province variable model, and full model), to select the best fit model, the widely applicable information criterion (WAIC) was used, which is best compared to the popular deviance information criterion (DIC) [15, 16]. The lowest WAIC is the best fit model. The results obtained from a given HMC analysis are not believed to be reliable until the chain has reached its stationary distribution and converged much more quickly [17, 18]. Therefore, to monitor the convergence diagnostics criteria, we used Rhat = 1, effective sample sizes (both Bulk_ESS and Tail_ESS) were greater than 1000, the time serious plots chains well mixed, and the density plot was smooth.

### 2.6 Ethical consideration

A waiver of written informed consent was secured from the International Review Board of Demographic and Health Surveys (DHS) program data archivists to download the dataset for this study.

## 3 Results

### 3.1 Characteristics of the study participants

A total of 89,091 under-five children were included for this study from the recent East African Countries DHS datasets. Overall, a total of 4,087 (5%) weighted number of under-five children were overweight and/or obese in East African Countries. More than 50% of the study participants were males. From all, near to 40% of under-five children were younger than two years. More than 30% of under-five children were severely or moderately anemic. More than 45% of under-five children live with poor household wealth status in East African Countries. Three-fourth of under-five children in East African countries live in rural inhabitant, and around 87% of them were found in the tropical area (Table 2).

**Table 2:** Characteristics of the study respondents and under-five children in East African Countries using the recent DHS dataset, 2021.

Variables		Frequency (n)		Percentage (%)
		Unweighted	Weighted	Weighted
Respondent age	No education	22,171	21,155	25.56
	Primary	41,010	41,759	50.45
	Secondary and above	20,009	19,860	23.99
Child sex	Male	44,950	44,770	50.25
	Female	44,648	44,321	49.75
Child age	≤ two years	35,759	35,616	39.98
	2–5 years	53,839	53,475	60.02
Child anemia status	Severe	1,174	1,202	2.15
	Moderate	15,395	15,668	28.04
	Mild	13,941	14,142	25.31
	Not anemic	24,490	24,866	44.50
House wealth status	Poor	42,827	40,798	45.79
	Middle	16,441	17,487	19.63
	Rich	30,330	30,806	34.58
Residence	Urban	22,776	21,134	23.72
	Rural	66,822	67,958	76.28
Altitude	Tropical	60,051	58,917	87.54
	Sub-tropical	5,229	6,433	9.56
	Cool	1,083	1,951	2.90
Country	Kenya	20,757	19,249	21.61
	Burundi	6,410	6,493	7.29
	Ethiopia	9,697	10,513	11.80
	Comoros	2,378	2,445	2.74
	Malawi	5,790	5,814	6.53
	Mozambique	9,296	9,423	10.58
	Ruanda	3,825	3,857	4.33
	Tanzania	10,301	9,942	11.16
	Uganda	5,286	5,243	5.89
	Zambia	9,694	9,705	10.89
	Zimbabwe	6,164	6,406	7.19
Country income	Low income	52,983	53,731	60.31
	Lower middle income	36,615	35,360	39.69
**Total**		**89,598**	**89,091**	**100**

### 3.2 Prevalence of overweight and/or obesity among under-five children

Overall, 4.59% (95 CI: 4.45–4.73) of under-five children in East African Countries were overweight and/or obese. Male (4.98) under-five children were more overweight and/or obese than females (4.19). The burden of under-five overweight and/or obesity increases among under-five children living in urban (5.75%) areas than rural (4.23%) areas. Under-five children living in lower-middle-income countries (4.69%) had high burden for overweight and/or obesity than children living in low-income countries (4.52%). The highest prevalence of overweight and/or obesity is found in Comoros (9%), followed by Rwanda (7.76%) and Mozambique (7.39). The lowest prevalence of overweight and/or obesity was reported from Ethiopia (2.83%), followed by Burundi (1.39%) (Table 3).

**Table 3:** Overweight or obesity among under-five children in East African Countries using the recent DHS dataset, 2021.

Variables		Weighted number of under-five children overweight and/or obese n (%)	Weighted sample size
Child sex	Male	2,231 (4.98)	44,770
	Female	1,857 (4.19)	44,321
Maternal education	No education	764 (3.61)	21,155
	Primary	1906 (4.56)	41,759
	Secondary and above	1207 (6.08)	19,860
Residence	Urban	1215 (5.75)	21,134
	Rural	2873 (4.23)	67,958
Household wealth status	Poor	1619 (3.97)	40,798
	Middle	773 (4.42)	17,487
	Rich	1695 (5.50)	30,806
Country income	Low income	2431 (4.52)	53,731
	Lower middle income	1657 (4.69)	35,360
Country	Kenya	792 (4.12)	19,249
	Ethiopia	297 (2.83)	10,513
	Burundi	90 (1.39)	6,493
	Comoros	220 (9.00)	2,445
	Malawi	263 (4.52)	5.814
	Mozambique	697 (7.39)	9,423
	Ruanda	299 (7.76)	3,858
	Tanzania	367 (3.69)	9,942
	Uganda	197 (3.75)	5,243
	Zambia	503 (5.18)	9,705
	Zimbabwe	362 (5.64)	6,406
**Status**	**Overweight and/or obese**	**4087 (4.59)**	**89,091**

### 3.3 Factors affecting under-five overweight and/or obesity

From Table 2 below, both the individual and region level variable model (model III) is the best fit model with the lowest WAIC (11851.10) when we compared it to the other models. Therefore, the effect size estimates in this model results were unbiased. This model has good convergence criteria with a sufficient sample size for bulk, tail greater than 1000, and Rhat vale of one. Therefore, all the results and interpretations were based on this model (model III).

Keeping all other variables constant, child age and sex, household wealth status, and country were associated with under-five children overweight and/or obese status.

Under-five children aged older than two years were 35% lower than having overweight and/or obese as compared to under-five children aged younger than two years (AOR = 0.65, 95% CrI: 0.57–0.73). Female under-five children had 16% less risk of developing overweight and/or obese as compared to male under-five children (AOR = 0.84, 95% CrI: 0.75–0.94). The odds of overweight and/or obesity among under-five children living in rich household wealth status were one-fourth higher than under-five children living in poor household wealth status (AOR = 1.25, 95% CrI: 1.06–1.49). Under-five children who live in Malawi, Mozambique, Rwanda, Tanzania, and Uganda have had a higher risk of under-five overweight and/or obese as compared to under-five children who live in Burundi (Table 4).

**Table 4:** Factors associated with under five overweight/obesity recent DHS report, East African Countries, 2021.

Variables	Null model	Model I	Model II	Model III
	AOR (95%CrI)	AOR (95%CrI)	AOR (95%CrI)	AOR (95%CrI)
**Child age**				
≤2 years		1		1
2–5 years		0.64 (0.57–0.72)		0.65 (0.57–0.73)
**Child sex**				
Male		1		1
Female		0.83 (0.75–0.93)		0.84 (0.75–0.94)
**Anemia status**				
Severe		1		1
Moderate		1.33 (0.88–2.07)		1.29 (0.84–1.99)
Mild		1.55 (1.01–2.49)		1.48 (0.99–2.27)
Not anemic		1.45 (0.94–2.35)		1.35 (0.89–2.09)
**Preceding birth interval**				
≥24 months		1		1
<23 months		1.02 (0.88–1.18)		1.03 (0.87–1.19)
**Birth order**				
≤ Three		1		1
> Four		1.04 (0.94–1.17)		1.04 (0.92–1.17)
**Maternal education**				
Unable to read and write		1		1
Primary education		1.04 (0.89–1.20)		1.00 (0.87–1.17)
Secondary and above		1.03 (0.84–1.25)		0.99 (0.79–1.24)
**Household wealth status**				
Poor		1		1
Middle		1.04 (0.89–1.19)		1.05 (0.89–1.23)
Rich		1.19 (1.05–1.37)		1.25 (1.06–1.46)
**Residence**				
Urban			1	1
Rural			0.95 (0.83–1.11)	1.15 (0.99–1.38)
**Country**				
Kenya			1	1
Burundi			0.97 (0.59–1.57)	-
Ethiopia			2.00 (0.74–5.77)	2.03 (0.70–5.96)
Comoros				-
Malawi			2.56 (1.51–4.34)	2.60 (1.49–4.51)
Mozambique			5.07 (3.39–7.47)	5.26 (3.52–7.79)
Ruanda			5.46 (3.33–8.59)	5.63 (3.46–9.08)
Tanzania			2.12 (1.48–3.07)	2.15 (1.47–3.12)
Uganda			2.53 (1.69–3.50)	2.62 (1.71–3.99)
Zambia			1.13 (0.43–3.20)	1.54 (0.44–4.89)
Zimbabwe			1.12 (0.43–3.12)	1.51 (0.44–4.81)
**Country income**				
Low income			1	1
Lower middle income			2.78 (0.99–7.23)	2.15 (0.68–7.34)
**Altitude**				
Tropical			1	1
Sub-Tropical			1.29 (1.03–1.59)	1.24 (0.96–1.56)
Cool			1.32 (0.85–1.98)	1.21 (0.75–1.88)
**ICC**	**12%**			
**WAIC**		**11875.43**	**11912.2**	**10728.6**

NB: Model I = Individual level factors, Model II = Community level factors, Model III = Full model, AOR = Adjusted Odds ratio, CrI = Credible Interval, ICC = Intraclass Correlation, WAIC = Widely Applicable Information Criteria.

## 4 Discussion

Obesity in the childhood period is a significant public health problem, and it is an indicator of severe conditions with premature morbidity and mortality. Childhood obesity increases the risk of all causes of mortality by three-fold in the early adulthood period [19].

This study revealed that the pooled prevalence of overweight and/or obesity among under-five children in East Africa was 4.59 % (95% CI = 4.45–4.73). This finding is higher than a study conducted in Maldives and Nepal [20]. While the finding of this study is lower than studies conducted in Ethiopia [14, 21, 22], Cameron (8%) [23], and sub-Saharan Africa (6.8%) [24]. The possible reason for this discrepancy might be socio-economic status, different feeding practices (cultural) among under-five children, methodological deference, cultural difference in feeding preferences, and nutrition assessment methods differences (using BMI z-score to assess overweight).

This study tried to investigate factors affecting the pooled prevalence of overweight and/or obesity among under-five children in East African Countries. Based on fully probabilistic analysis using the Bayesian approach, the age of under-five children significantly affects overweight and/or obesity status. Under-five children older than two years of age were 35% less likely to be overweight and/or obese than under-five children younger than two years of age. Previous studies conducted in Ethiopia [25], sub-Saharan Africa [24], and Cameron [23] findings were in line with this evidence. In developing countries, birth spacing is too short [26], which leads to early weaning for the child. Early weaning is a risk for rapid weight gain in infancy. So, this might be a cause of overweight/or obesity among children <2 years of age [27].

In this study, female sex and overweight and/or obesity of under-five children had a significant association. Female under-five children were 16% less likely to be overweight and/or obese than male under-five children. This finding is consistent with previous studies conducted in Ethiopia [28], sub-Saharan Africa [24], and Cameron [23]. The possible reasons will be biological deference (the difference in body composition during the fetal and postnatal period) [29], circulating leptin hormone among females [30], gender-based discrimination (early initiation of breastfeeding and complementary feeding practice for females) [31], and male children were highly involved on outdoor activities.

The higher household wealth affects under-five overweight and/or obesity. Under-five children born from rich households had a one-fourth risk of overweight and/or obesity as compared to children born from poor household wealth. Similarly, previous studies in Ethiopia [14], South Asia [20], sub-Saharan Africa [32], and Guangzhou, China [33] reported that children born from rich households were at high risk of overweight and/or obesity. In contrast to this, another study in sub-Saharan Africa evidenced that household wealth status had no significant effect on under-five obesity [24]. So, financial wellbeing in the household is associated with obesogenic behaviors (high intakes of calories rich food and physical inactivity) [34, 35]. Whereas this study evidenced that no significant association between country per capita income and overweight and/or obesity. That might be due to countries included in this study (lower-middle-income countries were three) and no significant heterogeneity as compared to low-income countries.

Furthermore, across countries, significant variation of under-five overweight and/or obesity was observed. Under-five children living in Malawi, Mozambique, Rwanda, Tanzania, and Uganda were more likely to be overweight and/or obese as compared to under-five children living in Kenya. Possibly this might be due to the sociocultural and feeding practice difference among under-five children across countries, universal health coverage, and country gross domestic product, in East African countries.

The strength of this study is the statistical analysis (Bayesian approach with a fully probabilistic model that used a baseline comparison), controls the dependency across region or province of the country by multilevel analysis, and this is big data analysis. Therefore, the evidence of the effect sizes will not be biased. In contrast, this study has the following limitations. First, there is no data for the other East African countries, this will not be generalizable for all countries in the region. Second, since the nature of demographic and health survey data is cross-sectional, it lacks casual evidence and is prone to social desirability bias.

## 5 Conclusion and recommendation

A significant number of under-five children were overweight and/or obese in the East African Countries. Older aged under-five children, male children, and children who live in rich household wealth were at high risk of had overweight and/or obesity. Also, under-five children who lived in Malawi, Mozambique, Ruanda, Tanzania, and Uganda are more likely to be overweight and/or obese in the region. So, stakeholders in the respective countries should have to give primary attention to control the alarming increment of overweight and/or obesity among under-five children. Even if developing countries were highly affected by undernutrition, currently, overweight and/or obesity is the main challenge. So, early intervention is warranted by these countries to halt the consequences of the double burden of malnutrition.

## 6 Ethical approval

Not applicable.

## 7 Consent for publication

Not applicable.

## Declarations

### Author contribution statement

Sofonyas Abebaw Tiruneh, Alemayehu Digssie Gebremariam, Melaku Tadege Engidaw, Desalegn Tesfa , Melkalem Mamuye Azanaw: Conceived and designed the experiments; Performed the experiments; Analyzed and interpreted the data; Wrote the paper.

Fentaw Teshome Dagnaw, Edgeit Abebe Zewde: Analyzed and interpreted the data; Wrote the paper.

### Funding statement

This research did not receive any specific grant from funding agencies in the public, commercial, or not-for-profit sectors.

### Data availability statement

Data associated with this study has been deposited at DHS (https://dhsprogram.com).

### Declaration of interests statement

The authors declare no conflict of interest.

### Additional information

No additional information is available for this paper.
